# Impact of Four Years of Annual Mass Drug Administration on Prevalence and Intensity of Schistosomiasis among Primary and High School Children in Western Kenya: A Repeated Cross-Sectional Study

**DOI:** 10.4269/ajtmh.17-0908

**Published:** 2018-03-12

**Authors:** Bernard O. Abudho, Eric M. Ndombi, Bernard Guya, Jennifer M. Carter, Diana K. Riner, Nupur Kittur, Diana M. S. Karanja, W. Evan Secor, Daniel G. Colley

**Affiliations:** 1 Centre for Global Health Research, Kenya Medical Research Institute, Kisumu, Kenya;; 2 Department of Biomedical Sciences, School of Public Health, Maseno University, Maseno, Kenya;; 3 Department of Pathology, Kenyatta University, Nairobi, Kenya;; 4 Center for Tropical and Emerging Global Diseases, University of Georgia, Athens, Georgia;; 5 Division of Parasitic Diseases and Malaria, Centers for Disease Control and Prevention, Atlanta, Georgia,; 6 Department of Microbiology, University of Georgia, Athens, Georgia

## Abstract

Schistosomiasis remains a major public health problem in Kenya. The World Health Organization recommends preventive chemotherapy with praziquantel (PZQ) to control morbidity due to schistosomiasis. Morbidity is considered linked to intensity of infection, which along with prevalence is used to determine the frequency of mass drug administration (MDA) to school-age children. We determined the impact of annual school-based MDA on children across all primary and high school years using a repeated cross-sectional study design in five schools near Lake Victoria in western Kenya, an area endemic for *Schistosoma mansoni*. At baseline and for the following four consecutive years, between 897 and 1,440 school children in Grades 1–12 were enrolled and evaluated by Kato-Katz for *S. mansoni* and soil-transmitted helminths (STH), followed by annual MDA with PZQ and albendazole. Four annual rounds of MDA with PZQ were associated with reduced *S. mansoni* prevalence in all school children (44.7–14.0%; *P* < 0.001) and mean intensity of infection by 91% (90.4 to 8.1 eggs per gram [epg] of stool; *P* < 0.001). Prevalence of high-intensity infection (≥ 400 epg) decreased from 6.8% at baseline to 0.3% by the end of the study. Soil-transmitted helminth infections, already low at baseline, also decreased significantly over the years. In this high prevalence area, annual school-based MDA with high coverage across all Grades (1–12) resulted in rapid and progressive declines in overall prevalence and intensity of infection. This decrease was dramatic in regard to heavy infections in older school-attending children.

## INTRODUCTION

Human schistosomiasis is a snail-transmitted trematode infection caused by any of five species in the genus *Schistosoma*. Globally, approximately 700 million people are at risk of this infection.^1,2^ More than 240 million people in 78 countries are estimated to be infected with schistosomes. More than 90% of the cases occur in sub-Saharan Africa, where the infection is estimated to cause more than 200,000 deaths annually.^3,4^ In Kenya, both *Schistosoma mansoni* and *Schistosoma haematobium* remain serious public health concerns with approximately 6 million people infected and an additional 15 million being at high risk of the infection in endemic areas of the country.^5^ *Schistosoma mansoni* infection is common in the western part of Kenya and its prevalence shows an inverse relationship with the distance from Lake Victoria.^6,7^

Schistosomiasis-associated morbidity and mortality reduction through treatment of school-age children was emphasized in 2001 when the World Health Assembly (WHA) 54.14 formally recognized the global burden of the infection. Mass drug administration (MDA) with praziquantel (PZQ) is the strategy the World Health Organization (WHO) presently recommends^8^ to reduce cumulative morbidity associated with schistosomiasis in endemic areas.^1,9–12^ When initiated, this strategy usually involves yearly or biennial (MDA) with PZQ to treat schistosomiasis and albendazole (ALB) to treat soil-transmitted helminths (STHs) in primary schools.^9^ The effectiveness of MDA programs for *S. mansoni* is mainly monitored by measuring changes in the prevalence of infection and drug treatment coverage, although WHO guidelines are also based on the prevalence of heavy infection (≥ 400 eggs per gram [epg] of feces).^13^ The aim of this school-wide, repeated, cross-sectional study was to evaluate the impact of 4 years of high-coverage annual MDA on the prevalence and intensity of *S. mansoni* infection across all grades of primary and secondary school children. The information generated from this study will add systematic data to the understanding on how these key indicators are impacted by yearly MDA over a period of 5 years.

## MATERIALS AND METHODS

### Ethics statement and eligibility criteria.

Ethical clearance was obtained from the Departmental and Institutional Scientific Steering Committees of Kenya Medical Research Institute (KEMRI) followed by the National KEMRI Scientific and Ethical Review Unit. The Institutional Review Board of the University of Georgia also reviewed and approved the study protocol (ID no. 00003501). On review of the protocol, the Centers for Disease Control and Prevention staff were not considered to be engaged with human subjects. Before the start of the study, informational meetings were held with the teachers, children, and parents of the children in the schools in the study. In these meetings, individual informed consent was first obtained from the parents or guardians of school-going children. Assent to participate in the study was also obtained from each participating child.

### Study area and population.

**Figure 1.**
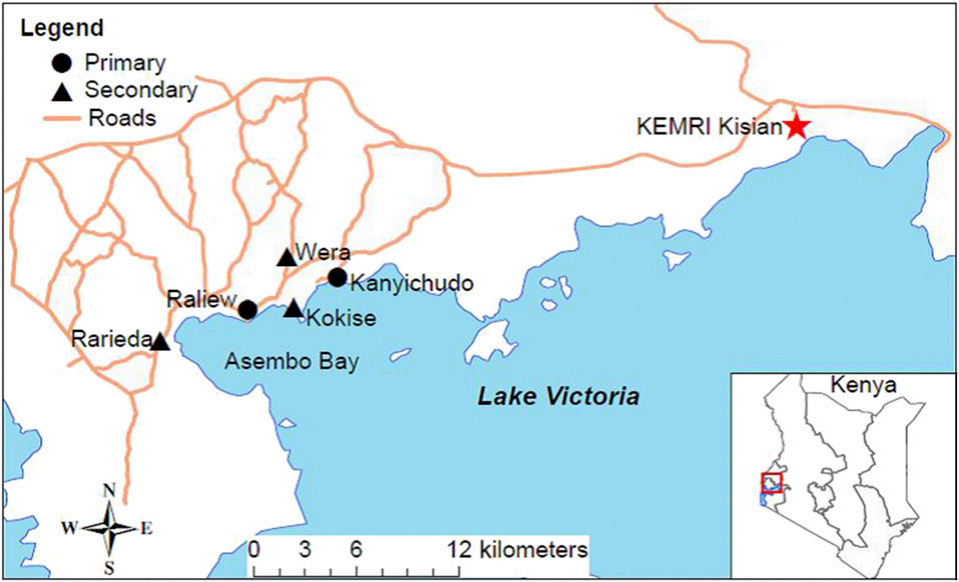
Map of the study site schools in the Asembo Bay area of western Kenya. This figure appears in color at www.ajtmh.org.

This study was conducted in two primary and three secondary schools in fishing villages located within 3 km of Lake Victoria in the Asembo Bay area of Rarieda Sub-county, Nyanza Region in western Kenya. The selected primary schools are the main feeder schools for those attending the selected secondary schools (Figure 1), and all were selected based on their expected prevalence of *S. mansoni* infection based on previous studies in the area.^6,11^ Approximately 96% of the population in this area is of the Luo ethnic group and the major occupations are fishing and subsistence farming.^14^ The area is highly endemic for *S. mansoni* as reported by a survey in primary schools in the area in 2001, which found that prevalence of *S. mansoni* ranged from 35% to 85%.^6^ More recent findings^11,15^ also support these high rates of infection in the region. The target population in the present study included all male and female children attending the five selected schools.

### Study design.

The study involved providing annual MDA to all children attending the five selected schools and doing an annual parasitologic survey of a randomly selected subset of children from all grades in each of the five schools before each annual MDA, using a repeated cross-sectional study design.

### Parasitologic surveys.

Children in each of the 12 grades were recruited and asked to provide fresh stool samples for *S. mansoni* and STH infection assessments. At baseline, only one stool sample was collected and two slides made using the Kato-Katz thick smear technique.^16^ In subsequent years (Years 1–4), three stool samples were collected on three consecutive days per child and two slides were made from each sample. The samples were transported to the KEMRI laboratory in Kisian where the Kato-Katz thick smears were prepared^16^ and examined for parasites’ eggs by two independent microscopists. Discrepancies were reconciled by a third experienced quality control microscopist. The presence of other helminth eggs (hookworms, *Ascaris lumbricoides*, and *Trichuris trichiura*) was recorded as either positive or negative, but quantitative counts of eggs were performed only for *S. mansoni*. The intensity of *S. mansoni* infection was expressed as epg by multiplying the arithmetic mean of egg counts from the total slides per child by 24 and categorized according to WHO guidelines as light (1–99 epg), moderate (100–399 epg), and heavy (≥ 400 epg).^13,17^

### Mass drug administration.

After baseline stool examinations and immediately after subsequent yearly stool surveys for the next 4 years, all children attending the five selected schools were offered treatment with PZQ (40 mg/kg) and ALB (400 mg). Drug administration was by directly observed therapy conducted by trained members of the study team. Normal school attendance reported by school health teachers and head teachers was typically more than 90%. Extensive publicity efforts were used to promote each annual MDA through provincial administrators, church leaders, and parents, among other community members. As a result, attendance on MDA days was exceptionally high, resulting in very high annual treatment coverage rates in the five schools.

### Data management and analysis.

The latitude and longitude coordinates of each school were obtained using a Global Positioning System on Android phones and projected to universal transverse mercator zone 36S. Maps were created with ArcGIS (version 10.3; ESRI, Inc., Redlands, CA). Each study participant was assigned a unique identification number. Data were entered using Microsoft Excel and kept confidential. Data were analyzed using IBM SPSS version 24 (Armork, NY) and GraphPad Prism version 6 (La Jolla, CA). Mann–Whitney nonparametric analysis was used for evaluating infection intensity across years. A χ^2^ test for trend (Cochran–Armitage) was used to evaluate the association between annual treatments and changes in prevalence of *S. mansoni* and STH. Tests were considered statistically significant at *P* < 0.05.

## RESULTS

### Mass drug administration and study populations.

Figure 1 is a map of the Asembo Bay study area and the locations of the study schools in relationship to Lake Victoria. Annual school-wide preventive chemotherapy with PZQ and ALB was provided in all five schools for 4 years. As a result of the extensive sensitization of the surrounding communities and the schools, treatment coverage exceeded 90% each year. At baseline, a total of 1,110 children from the two primary schools and three secondary schools were enrolled for the parasitologic study. In subsequent years, 897 (Year 1), 1,302 (Year 2), 1,327 (Year 3), and 1,440 (Year 4) children were evaluated for *S. mansoni* and STH infections. On average, 100 children (range = 31–202) were randomly enrolled from each grade (Grades 1–12) other than Year 1 when an error occurred and no first grade students were evaluated (Table 1). Although many children in each grade were evaluated each year, the proportion represented in each grade shifted somewhat, resulting in more students being evaluated in lower grades at baseline and more being evaluated in the upper grades by Years 2, 3, and 4. The proportion of male students varied from 49% to 57% over the years.

**Table 1:** Total number of children screened from each grade in all years

Grade	Baseline, *N* (%)	Year 1, *N* (%)	Year 2, *N* (%)	Year 3, *N* (%)	Year 4, *N* (%)
1	124 (12.3)	0 (0)	77 (5.9)	71 (5.4)	70 (4.9)
2	111 (11.0)	72 (8.0)	80 (6.1)	129 (9.7)	80 (5.6)
3	126 (12.5)	81 (9.0)	88 (6.8)	100 (7.5)	98 (6.8)
4	128 (12.7)	108 (12.0)	111 (8.5)	111 (8.4)	93 (6.5)
5	90 (8.9)	83 (9.3)	103 (7.9)	90 (6.8)	94 (6.5)
6	84 (8.3)	86 (9.6)	103 (7.9)	80 (6.0)	104 (7.2)
7	79 (7.8)	97 (10.8)	126 (9.7)	108 (8.1)	105 (7.3)
8	65 (6.4)	65 (7.2)	62 (4.8)	76 (5.7)	88 (6.1)
9	63 (6.2)	39 (4.3)	156 (12.0)	177 (13.3)	202 (14.0)
10	49 (4.8)	122 (13.6)	200 (15.4)	181 (13.6)	193 (13.4)
11	61 (6.0)	70 (7.8)	124 (9.5)	105 (7.9)	168 (11.7)
12	31 (3.1)	74 (8.2)	72 (5.5)	99 (7.5)	145 (10.1)
Total	1,011 (100)	897 (100)	1,302 (100.0)	1,327 (100.0)	1,440 (100.0)

### Effects of MDA on *S. mansoni* and STH infections.

**Figure 2.**
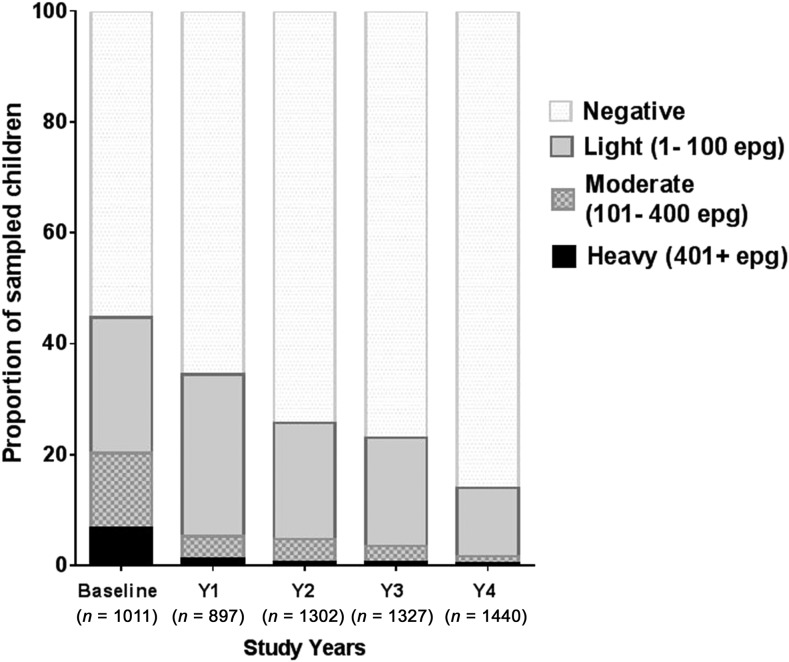
Overall prevalence and intensity (by World Health Organization standard intensity categories) of *Schistosoma mansoni* infection for school children in the primary and secondary schools studied.

The overall prevalence of *S. mansoni* was 44.7% at baseline and decreased after each round of MDA (Table 2). Each of the five schools declined in *S. mansoni* prevalence by more than 60% after the 4 years of MDAs. The prevalence of hookworms and *A. lumbricoides* was approximately 3% at baseline and rapidly decreased after annual MDA with ALB to < 1% for both infections by year 4 (Table 2). *Trichuris trichiura* prevalence, which was 5.3% at baseline, decreased more gradually to 1.3% at Year 4 (Table 2). Analyses of the yearly progression of the changes in prevalence for these helminths by the Cochran–Armitage test for trend indicate that prevalence of each infection changed significantly over the study years (*S. mansoni*, χ^2^ = 311.2, *P* < 0.0001; hookworm, χ^2^ = 54.9, *P* < 0.0001; Ascaris, χ^2^ = 10.6, *P* < 0.0001; and *Trichuris*, χ^2^ = 28.9, *P* < 0.0001). Figure 2 demonstrates the yearly progressive decrease of both prevalence and intensity of *S. mansoni* infection for all students, resulting in almost virtual elimination of heavy infections (from 6.8% to 0.3%) after four rounds of school-based MDA.

**Table 2:** Overall prevalence of *Schistosoma mansoni* and soil-transmitted helminths among screened primary and secondary students over 5 years

	Baseline (*N* = 1,011), %	Year 1 (*N* = 897), %	Year 2 (*N* = 1,302), %	Year 3 (*N* = 1,327), %	Year 4 (*N* = 1,440), 5
*S. mansoni*	44.7	34.4	25.7	23.1	14.0
Hookworms	3.0	0.1	0.6	0.2	0
*Ascaris lumbricoides*	2.8	0	1.8	1.1	0.6
*Trichuris trichiura*	5.3	4.3	4.4	3.4	1.3

**Figure 3.**
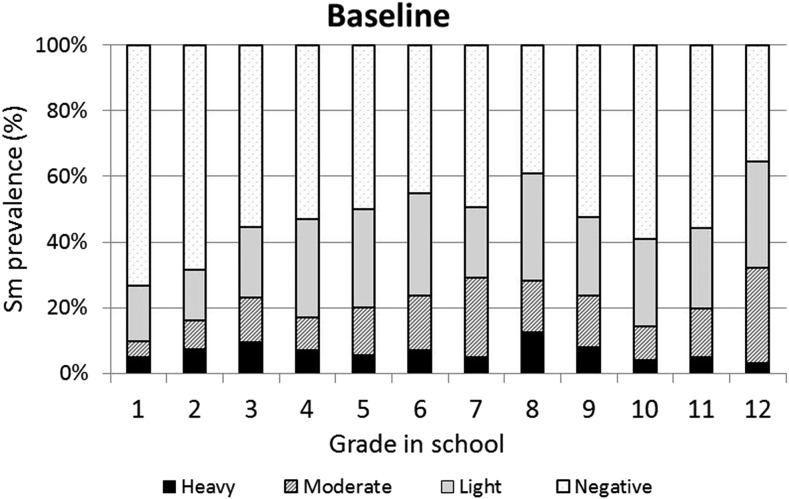
Baseline *Schistosoma mansoni* grade vs. prevalence/intensity curves.

**Figure 4.**
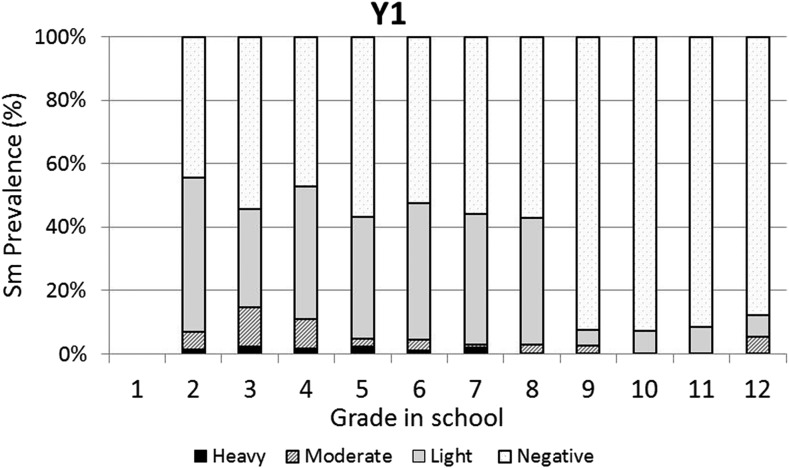
Year 1 *Schistosoma mansoni* Grade vs. prevalence/intensity curves.

**Figure 5.**
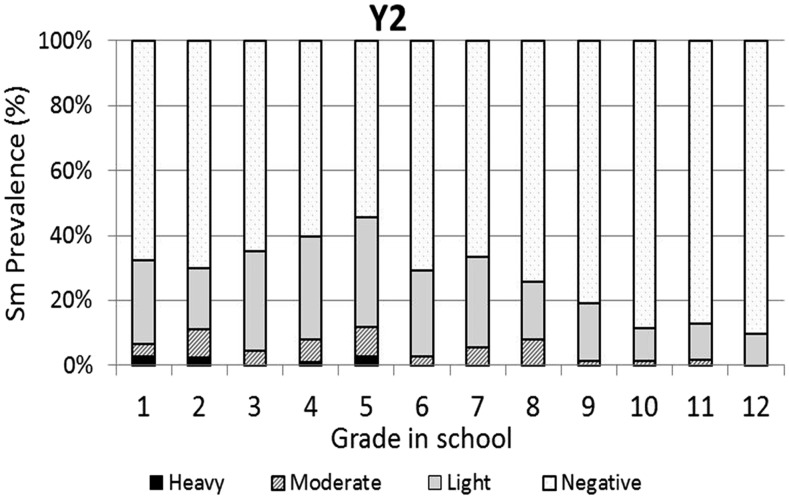
Year 2 *Schistosoma mansoni* Grade vs. prevalence/intensity curves.

**Figure 6.**
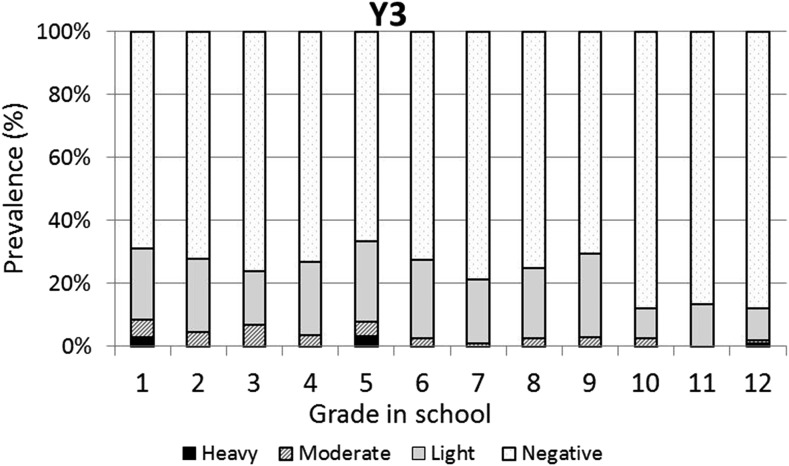
Year 3 *Schistosoma mansoni* Grade vs. prevalence/intensity curves.

**Figure 7.**
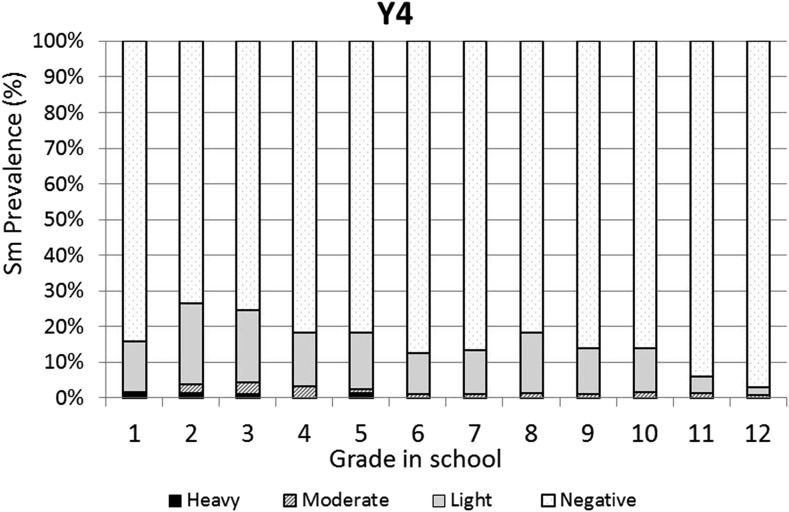
Year 4 *Schistosoma mansoni* Grade vs. prevalence/intensity curves.

The baseline infection data across grades were consistent with typical schistosomiasis age/prevalence curves where prevalence increases with age (Figure 3). However, the same curve was not observed for the prevalence of heavy infections, which varied across all grades with no discernible pattern. One year after the first round of high-coverage MDA, prevalence was decreased and infections levels were dramatically shifted from heavy or moderate to light, with very low prevalence of heavy infections in the lower grades (1–7) and no heavy infections in the older students (Grades 8–12; Figure 4). This pattern was essentially repeated in subsequent years. After two rounds of MDA (Year 2, Figure 5), the age/prevalence pattern was shifted compared with baseline with high prevalence and heavy infections present only in the lower grades. This pattern was maintained after three rounds of MDA (Year 3, Figure 6) but became less obvious after the fourth round of MDA (Year 4, Figure 7) because prevalence and intensity were decreased in all grades and the curves became essentially flat.

Table 3 summarizes the proportions of heavy infections by grade and year after MDA. Although nearly all grades were higher than the WHO threshold of 5% heavy infections at baseline, one round of MDA was sufficient to achieve no or extremely low proportions of heavy infections, especially in the older students. This decrease in heavy infections was reflective of the overall change in intensity, which at baseline was 90.4 epg, and after four rounds of MDA was 8.1 epg. This was a statistically significant (*P* < 0.001) 91% mean reduction in overall intensity.

**Table 3:** Prevalence (%) of heavy infections (≥ 400 epg) among screened children over 5 years of study

Grade	Baseline	Year 1	Year 2	Year 3	Year 4	*P* value for χ^2^ test for trend
1	4.8	–	2.6	2.8	1.4	0.184
2	7.2	1.4	2.5	0	1.3	0.002*
3	9.5	2.5	0	0	1.0	< 0.001*
4	7.0	1.9	0.9	0	0	< 0.001*
5	5.6	2.4	2.9	3.3	1.1	0.149
6	7.1	1.2	0	0	0	< 0.001*
7	5.1	2.1	0	0	0	0.0011*
8	12.5	0	0	0	0	< 0.001*
9	7.9	0	0	0	0	< 0.001*
10	4.1	0	0	0.6	0.5	0.239
11	4.9	0	0	0	0	0.002*
12	3.2	0	0	1.0	0	0.234

epg = eggs per gram. Grades/Years with ≥ 5% prevalence of heavy infection are highlighted in dark grey. Grades/Years with 1–5% prevalence of heavy infection are highlighted in light grey.

* Indicates statistical significance.

## DISCUSSION

Baseline data from the present study of primary and secondary school children indicate that *S. mansoni* infection was prevalent among untreated school children in the Asembo Bay area of western Kenya. Although almost half of the children were infected with *S. mansoni*, most had light to moderate intensities of infection. In general, fewer than 10% were heavily infected (≥ 400 epg). Presently, WHO guidelines recommend annual school-based MDA with PZQ in areas where prevalence of infection is ≥ 50%. Where the prevalence of infection is less than 50% but at or more than 10%, current recommendations call for school-based MDA every other year, whereas if prevalence is less than 10% infection, MDA twice during primary school is considered sufficient.^13^ In addition, the prevalence of heavy infection is recommended by WHO as a guide for when to move from morbidity control to elimination as a public health problem and then on to elimination of transmission. When the prevalence of heavy infection is ≥ 5% in sentinel sites, morbidity control is considered to be still in order, and countries would be eligible for elimination as a public health problem when heavy infection prevalence is less than 1% in all sentinel sites.^13^ The data presented here regarding heavy infections in school children may contribute to potential re-evaluations of some of these targets, guidelines, and goals.

Mass drug administration with PZQ and ALB was used in this school-based program to treat school children for schistosomiasis and STH infections, respectively. After one round of MDA with PZQ, prevalence of heavy infection was rapidly reduced in all the grades and driven to essentially zero in Grades 8–12. Two and three rounds of PZQ MDA also progressively continued to reduce both prevalence and intensities to lower levels, with most grades exhibiting low proportions of heavy infections (Figures 4 and 5). Four rounds of annual PZQ MDA significantly reduced *S. mansoni* infection prevalence and virtually eliminated heavy infections as defined by WHO guidelines, supportive of the goal to prevent high-intensity schistosome infections that are generally associated with morbidity, including anemia in children.^13,18,19^ Prevalence levels of STH infections were quite low at baseline compared with a 2003 report from western Kenya.^6^ However, a single round of ALB MDA was conducted in Kenyan schools in 2009, and our data are consistent with more recent surveys of STH infection levels in this area.^20–22^

Another observation made possible by our Grade-by-Grade study design and analysis indicates that by Year 4, on average, children in the lower grades had somewhat higher levels of heavy infections than their counterparts in Grades 6–12. There could be several explanations for this observation: 1) Because treatment is school based, those in the lower, entering grades did not receive as many rounds of MDA as those in the upper grades; 2) Those in the higher grades do not frequent active transmission sites as often as the younger children and would thus have a lower risk of reinfection; 3) Those in the higher grades, because of more opportunities to experience dying (or PZQ-killed) adult worms^23,24^ had achieved some level of immune-mediated resistance to reinfection; and/or 4) Those in the higher grades perhaps benefited more from health education and behavioral change that may have accompanied the sensitization and education associated with MDA. These possibilities are not mutually exclusive and it could be any combination that led to lower levels of reinfection in older children. Even though mean epg counts decreased by more than 90% and heavy-intensity infections were almost eliminated, four rounds of high-coverage annual school-based MDA was not sufficient to eliminate schistosomiasis. For areas where elimination of transmission may be considered feasible, MDA will need to be complemented with other schistosomiasis-integrated control programs such as provision of clean water, provision of improved sanitation, health education, and/or snail control.^25^

Incoming first year students would not have received an annual MDA because they were not previously enrolled in primary school. They could therefore be considered a “nontarget population” on entering primary school. It is interesting to note that in Year 4, when this entry, “nontarget” group is compared with this same group at baseline, they showed a decrease in prevalence (26.5–15.7%) and prevalence of heavy infection (4.5–1.4%). These changes did not achieve statistical significance but could be taken as an encouraging observation that might suggest an overall reduction in transmission in the area, perhaps due to a lower number of eggs being excreted in the environment after four rounds of MDA. Sensitization in the form of recommended behavior changes and health education, which took place before each MDA, might also have played a role in altering fecal contamination of the environment and exposure to cercariae.^26^

Although the present study demonstrates the ability of annual school-based MDA to lower the proportion of infected children from 44.7% at baseline to 14.0% in Year 4, the study was not designed to evaluate changes in MDA strategies. Such studies are being performed, in the same province as this study, and will perhaps assist in determining which MDA strategies will be most effective in maintaining or advancing the types of outcome seen in the present study.^26,27^

Limitations in the execution of this study include screening of only one stool sample with two Kato-Katz slides at baseline as opposed to three consecutive stool samples of two slides each in the following years. Although this is what is routinely done by control programs, in this study, it may have underestimated the initial prevalence and intensities of infection at baseline as compared with the later time points. In Year 1, we had a protocol deviation that led to the failure to collect stool samples from first year, Grade 1 students.

These results show that *S. mansoni* infection was prevalent among children in the five schools studied in the Asembo Bay region of western Kenya. Mass drug administration with PZQ had an overall positive impact on the levels of *S. mansoni* infection in both primary and secondary school children. Thus, when assiduously applied, preventive chemotherapy, as supported by WHO guidelines, offers a practical program to control *S. mansoni* infection and its associated morbidity.^28–30^ However, the findings from this study also indicatethat additional schistosomiasis interventions will likely be needed to complement MDA to achieve the goal of elimination promoted by WHA resolution 65.21.^31^ Until other control measures are practical for field deployment, morbidity control through school-based MDA with PZQ still needs to be the primary emphasis in schistosomiasis control in sub-Saharan Africa.
